# Socio-cultural and personal profile of international medical students in Tunisia

**DOI:** 10.1192/j.eurpsy.2025.1284

**Published:** 2025-08-26

**Authors:** N. Bouattour, F. Guermazi, M. Lalaoui Rhali, R. Masmoudi, I. Baâti, J. Masmoudi

**Affiliations:** 1 Psychiatry A, Hedi Chaker University Hospital, Sfax, Tunisia

## Abstract

**Introduction:**

Foreign medical students in Tunisia represent a dynamic academic community, making an essential contribution to the global medical training. Their presence creates a vibrant academic ecosystem, conducive to discovery, intercultural exchange and medical innovation.

**Objectives:**

To determine the socio-cultural and the personal profile of foreign medical students, in order to optimize their academic performance and facilitate access to a high-quality professional training.

**Methods:**

We conducted a cross-sectional, descriptive and analytical study, from July to September 2023 among foreign students at the Sfax Faculty of Medicine via an anonymous self-questionnaire via “Google Forms” shared among students through social media. We used scale of satisfaction in studies, which is a 5-item scale, each rated on a 7-point Likert scale indicating the degree of agreement or disagreement with the statements.

**Results:**

Seventy-two foreign student completed the survey. The average age was 25 ± 3.45 years. Students of Moroccan origin accounted 68.1%. The socioeconomic status was average in 93.1%. The majority of respondents were single (77.8%). One-third of the students lived with a roommate. Fifty-one per cent of the students had at least one family member in the healthcare field. Of those surveyed, 61.1% were enrolled in the third cycle of medical studies, 26.5% were enrolled in the second cycle of medical studies and 12.5% were enrolled in the first cycle of medical studies. The average length of stay in Tunisia was 6.5 years. Seventy-seven percent were satisfied with the choice of Tunisia for the medical studies. Seventy-nine percent of the students were satisfied with their academic performance. Coffee was the most consumed substance (88.9%) followed by tobacco (47.2%), alcohol (36.1%) and illicit drugs (26.7%). Fifty-nine percent (N=41) practiced sports and 16.7% had an artistic leisure activity. We found a statistical association between satisfaction and the practice of a leisure activity (p=0.007) and a statistical negative association with having a family member in the healthcare field (p=0.022). We found no statistical association with the choice of Tunisia as a destination even though the mean score was higher among the students that was satisfied with their choice. The quality of the studies in Tunisia as well as the geographic localization were the main reasons for choosing Tunisia No statistically significant association was found with gender, socioeconomic status or marital status, substance consumption.

**Conclusions:**

The medical field studies are still challenging for students in general due heavy learning process and training process adding to that the personnel, cultural changes that a foreign student can face. Highlighting these aspects can contribute to improving their experience, by focusing on the learning process as well as making time to practice their leisure activities in order to break the routine.

**Disclosure of Interest:**

None Declared

